# Microtiter plate with built-in oxygen sensors: a novel approach to investigate the dynamics of *Pseudomonas aeruginosa* growth suppression in the presence of divalent cations and antibiotics

**DOI:** 10.1007/s00203-022-02877-y

**Published:** 2022-05-04

**Authors:** Wafa Almatrood, Ismini Nakouti, Glyn Hobbs

**Affiliations:** Centre for Natural Products Discovery (CNPD), School of Pharmacy and Biomolecular Sciences, Liverpool John Moores University, Liverpool, L3 3AF UK

**Keywords:** *Pseudomonas aeruginosa* PA01, OxoPlate OP96U, Antibiotics, Ca^2+^, Mg^2+^

## Abstract

The depletion of dissolved oxygen in a defined synthetic medium can be measured in real time, using a micro-well plate format, associated with a fluorescent plate reader. This technology is appropriate for investigating the effect of antibiotics on cell kinetics because there is a direct correlation between the latter and the amount of dissolved oxygen in the medium of an assay. In this study, the metabolic activity of the opportunistic human pathogen *Pseudomonas aeruginosa* PA01 was investigated using the OxoPlate OP96U optical sensor technology. The response of *P. aeruginosa* to aminoglycoside antibiotics when Ca^2+^and Mg^2+^ ions are present in the Evans defined synthetic medium was measured. The results revealed that the effect of antibiotics on *P. aeruginosa* is influenced by the concentration of divalent cations present in the test medium, although the efficiency of Ca^2+^ in supressing antibiotic activity was found to be greater than that of Mg^2+^. By comparison to tobramycin, the effect of amikacin is largely inhibited by the Ca^2+^and Mg^2+^concentrations. The study results underscore that the reliability of the observation of growth inhibitors is enhanced by the oxygen consumption measurements. Thus, the OxoPlate OP96U system is proven to be an accurate method to test the effectiveness of antibiotic treatments against *P. aeruginosa*.

## Introduction

Antibiotic resistance has become more and more widespread, and is now considered to be among the gravest threats to hospitals and the wider community (Rice 2009). The rapid speed of resistance evolving throughout the bacterial population is the greatest risk to effective bacterial infection treatment (Cars et al. 2011). Multiple drug resistant strains of *Pseudomonas aeruginosa* are now common, making the treatment of resultant infections increasingly problematic. *P. aeruginosa* exhibits the highest variation in drug susceptibility among the wide range of bacterial species on which the ionic composition of the test medium was demonstrated to have an influence (Fass and Barnishan 1979). According to Brogden et al. (1976), considerable discrepancies in the ion concentrations can generate different Minimum Inhibitory Concentration (MIC) values, which could determine the use of an inadequate antibiotic class against infections with *Pseudomonas*. As such, it is important to focus on the development of technologically advanced systems for identifying the effectiveness of antibiotic treatments against resistant bacterial infections.

The reliability of the susceptibility tests used to evaluate the antimicrobial activities of antibiotics against bacteria is considered an important parameter for the sustainable use of antimicrobials. The effects of antibiotics are typically observed, using traditional techniques, such as examining their inhibitory impact on the organisms’ growth. In standard methods (endpoint or off-line measurement), micro-broth dilution method (Felegle et al. 1979; Ostenson et al. 1977; Zuravleff et al. 1982), microscopy (Koch 1994) and visual counting based on colony growth on agar plates (Sahalan et al. 2013) are the main tools for bacterial quantification. Despite some degree of success, the use of automated spectrophotometry to measure turbidity may produce incorrect results due to condensation and microtiter plate interferences. Although accuracy can be improved using cell staining or redox reagents (e.g. resazurin), these methods involve multiple stages while the samples may be damaged by the reagent additions (Teethaisong et al. 2017). Assays that employ whole cells are difficult to adapt to high throughput applications (Desnottes 1996). Using the above methods, the dynamics of microbial growth in the presence of antibiotics cannot be monitored with high temporal resolution. Thus, assays able to detect or quantify the growth-inhibition activity of bacterial cells when exposed to antibiotics are urgently needed.

The measurement of oxygen consumption is central to numerous research areas since this substance is inevitably fundamental to aerobic cells’ behaviour and growth. This study proposes a new fluorescent assay that measures the metabolic activity of cells (i.e. capacity for oxygen consumption) as a way of determining bacterial growth. The fluorescence intensity of the oxygen sensor is correlated with the level of oxygen consumption and the general level of cellular respiration. Measuring dissolved oxygen in microtiter plates is useful for screening of drugs (Wesolowski et al. 2008, 2010) and enzymes that consume oxygen such as oxidases, aerobic cell activities, and pollutant biological deterioration, as well as for toxicity appraisals (Arain et al. 2006; West et al. 2014). It can also be used to detect the respiration of cells (Heller et al. 2012; Zagari et al. 2013; Edwards et al. 2015). The OxoPlate OP96U analyses samples with two fluorescent dyes, one being receptive to oxygen and the other emitting a regular signal. Any alterations in the otherwise constant ratio between these two dyes are recorded in real time. The measurement of oxygen depletion indirectly indicates the level of bacterial development which, alongside bacterial metabolic activity, alters the oxygen concentration with time. The OxoPlate system can be adjusted to suit the development pattern of a certain bacterium (Hutter and John 2004). This work employed the OxoPlate OP96U, not only to estimate the MIC, but also to distinguish between bactericidal and bacteriostatic effects. The advantages of this technique is the lack of requirement for calibration, ease of use, and real-time automation (Hutter and John 2004). Susceptibility assays are just one of the wide ranges of testing related to the rational use of antibiotics that OxoPlate OP96U is compatible with.

This study aims to provide novel insights surrounding the ability of 96-well microtiter plates (OxoPlate OP96U) with incorporated optical sensors of oxygen to determine the effect of aminoglycosides on *P. aeruginosa* PA01 in both the presence and absence of Ca^2+^ and Mg^2+^ ions in Evan’s defined synthetic medium. An in vitro experiment was conducted to shed more light on the observed fluctuations in the efficiency of aminoglycoside antibiotics in vivo.

## Materials and methods

### Bacterial strain and growth condition

The present study employed *P. aeruginosa* PA01 (ATCC 15,692) to evaluate the effect of aminoglycosides in conjunction with the presence/absence of Ca^2+^ and Mg^2+^ ions. The strain was cultivated on Evan’s media consisting of: NaH_2_PO_4_.2H_2_O (0.62 g/ l) (BDH, England), KCl (0.75 g/l) (Fluka), NaNO_3_ (0.849 g/l) (Sigma-Aldrich), Na_2_SO_4_ (0.28 g/l) (Sigma), citric acid (0.38 g/l) 0.02% (v/v) (BDH, England), glucose (1.8 g/l) (Thermo Fisher Scientific, UK), CaCl_2_.2H_2_O (0.037 g/l) (BDH, England), MgCl_2_ (0.11 g/l) (Amresco, United States American (USA)) and filter sterilised trace elements. The pH was adjusted to 7.2 prior to sterilisation by autoclaving. The concentrations of both Ca^2+^ and Mg^2+^ were amended to 1–5 mM when required.

Trace element solution contained ZnO (4.1 g/l), FeCl_3_ (3.2 g/l) (Sigma–Aldrich), MnCl_2_.4H_2_O (2.0 g/l), CuCl_2_ (1.4 g/l) (BDH, England), CoCl_2_.6H_2_O (4.8 g/l) (BDH, England), Na_2_MoO_4_.2H_2_O (0.0048 g/l) (BDH, England), NOB_4_O_7_.10 H_2_O (3.82 g/l) (Sigma, Japan) in 80 mL of concentrated HCl (Sigma–Aldrich, UK). Shaking flasks (250 ml) containing 50 mL of sterile Evan’s media and 50* μl* of filtrated trace elements were inoculated with *P. aeruginosa* and incubated overnight at 30 °C, 250 rpm.

### Determination of minimum bactericidal concentration (MBC)

MBCs (96-well plate) were determined based on the levels of oxygen consumption using the FLUOstar OPTIMA fluorescent plate reader (BMG LABTECH Ltd). Each well contained 180 μL of sterile Evan’s media, 10 μL of the specific antibiotic (0.3–2.5 μg/mL amikacin disulphate salt or 0.12–1 μg/mL tobramycin sulphate salt) and 10 μL of an overnight *P. aeruginosa* culture (0.5 McFarland standard in Evan’s media). Each assay was performed in triplicate. The positive and negative controls had the antibiotics omitted and replaced with either 10 μL of sterile water or 10 μL sodium hypochlorite (reagent grade chlorine 10–15%v/v, Sigma–Aldrich), respectively. The plate was incubated for 72 h at 30 °C**.**

### Effect of Ca^2+^ and Mg^2+^ on the kinetics of growth inhibition

To investigate how the dynamics of *P. aeruginosa* growth responded to the presence of Ca^2+^ and Mg^2+^ alongside bactericidal doses of the antibiotics (amikacin and tobramycin), different concentrations of Ca^2+^ and Mg^2+^ (1–5 mM) were incorporated into the Evan’s media. The approach involved an OxoPlate® (PreSens), which is a 96-well microtitre plate, containing specific sensors that measure oxygen levels in each well using a fluorescence plate reader (BMG/OPTIMA.). Each well contained 160 μL of Evan’s media (Ca^2+^ and Mg^2+^ ions omitted), 10 μL of the specific antibiotic, 20 μL ions (Ca^2+^ and Mg^2+^) to attain the necessary final concentrations and 10 μL of an overnight culture (0.5 McFarland standard). Six replicates were performed for each experiment and automatic fluorescent readings were obtained every 5 min. The plate was incubated for 72 h at 30 ºC in a horizontal shaking (100 rpm/ 5 min) fluorescent plate reader (BMG/OPTIMA). A polystyrene lid (Thermo Scientific, AB-0718) was used to cover the plate, to prevent gas permeating its adhesive membrane.

### OxoPlate measurements OxoPlate®/statistical analysis and antibacterial susceptibility test

The OxoPlate optical sensor OP96U (Precision Sensing GmbH, Germany) was employed. The core element of the OxoPlate optical sensor is a thin polymer film located at the bottom of each well measuring around 10 μm and comprising an indicator dye and a reference dye. The phosphorescence potency of the indicator dye (I-_indicator_) is determined by the amount of oxygen that the sample contains. By contrast, the ratio I_R_ of the oxygen volume, which denotes the oxygen concentration, does not affect the fluorescence potency of the reference dye (I-_reference_). The fluorescent plate reader was used to scan the optical sensors at the base of the OxoPlate wells. The test was undertaken using the time-resolve fluorescent measurement method and plate mode reading for slow kinetics. The plate was measured with an excitation filter 540/650 nm to determine the indicator dye and with an emission filter 540/590 nm to determine the reference dye. The MARS data analysis software version 2.20 obtained from the BMG LABTECH Ltd documented the results and exhibited them in the form of curves. To determine means, fluorescence intensity ratio and oxygen concentration, Microsoft Excel 2010 was used for data analysis. For graphical visualization, Sigma plot v.12.3 was used. The equations used to calculate the fluorescence intensity ratio and the oxygen concentration pO_2_ as percentage air saturation in each well were I_R_ = I _indicator_/I _reference_ and pO_2_% = 100. ((*K*_0_/I_R_-1) / (*K*_0_/*K*_100_-1)), respectively. Where *K*_0_ is the maximum value of I_R_ and *K*_100_ is the minimum value of I_R_.

## Results

### Deducing the MBC from OxoPlate measurements:

**Fig. 1:**
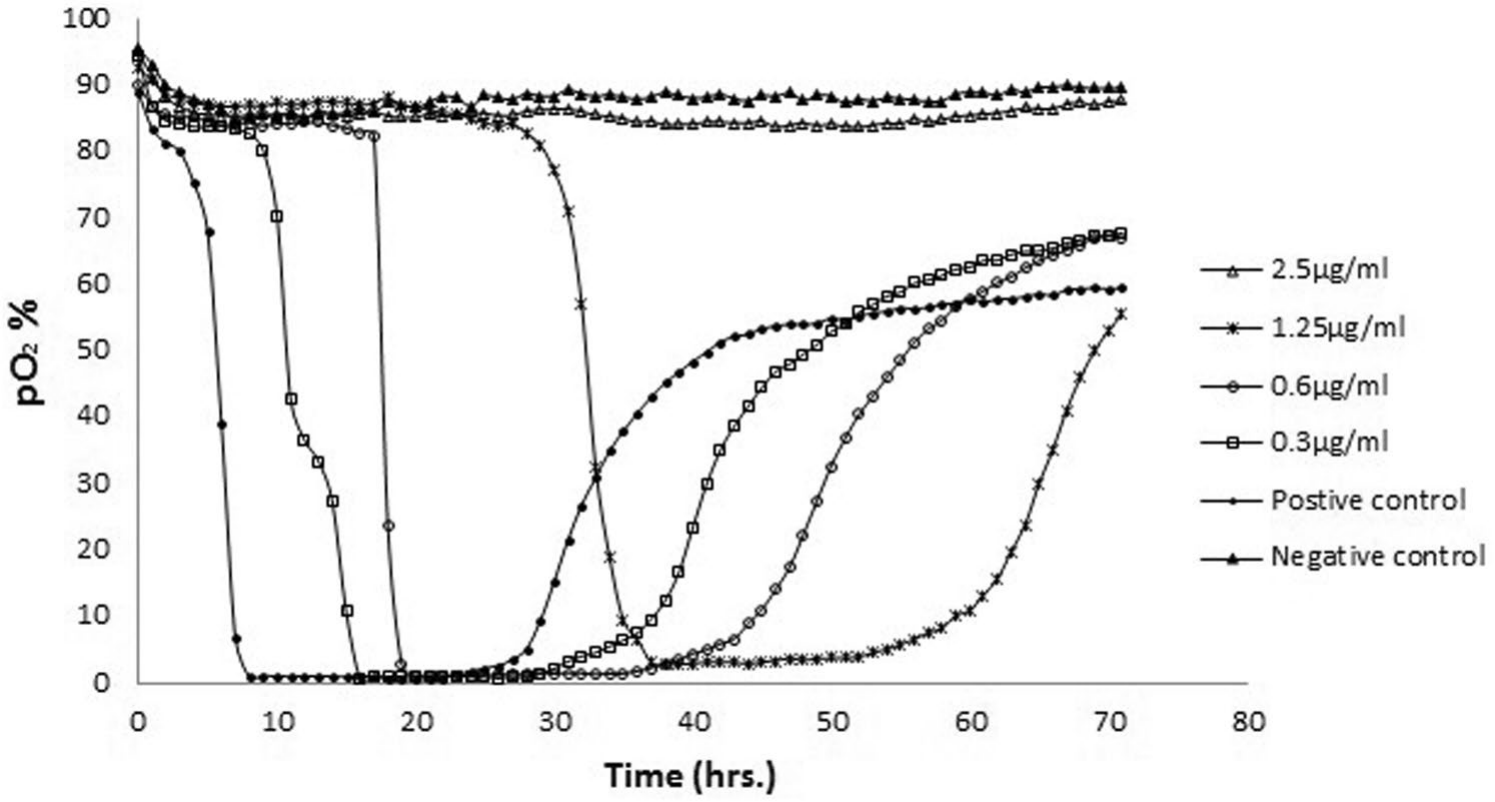
The MBC of amikacin against PAO1*.* The oxygen consumption of an overnight culture of planktonic cells of PAO1 treated with amikacin using a fluorescent plate reader incubated at 30 °C for 72 h. Antibiotic concentrations indicated. The MBC represents the time to detect the lowest concentration that induced an increase in the oxygen concentration as a result of oxygen diffusion back into the wells following cell death. In contrast to the untreated positive control sample, the negative control represents an overnight culture injected with a solution of sodium hypochorite (10% v/v)

**Fig. 2:**
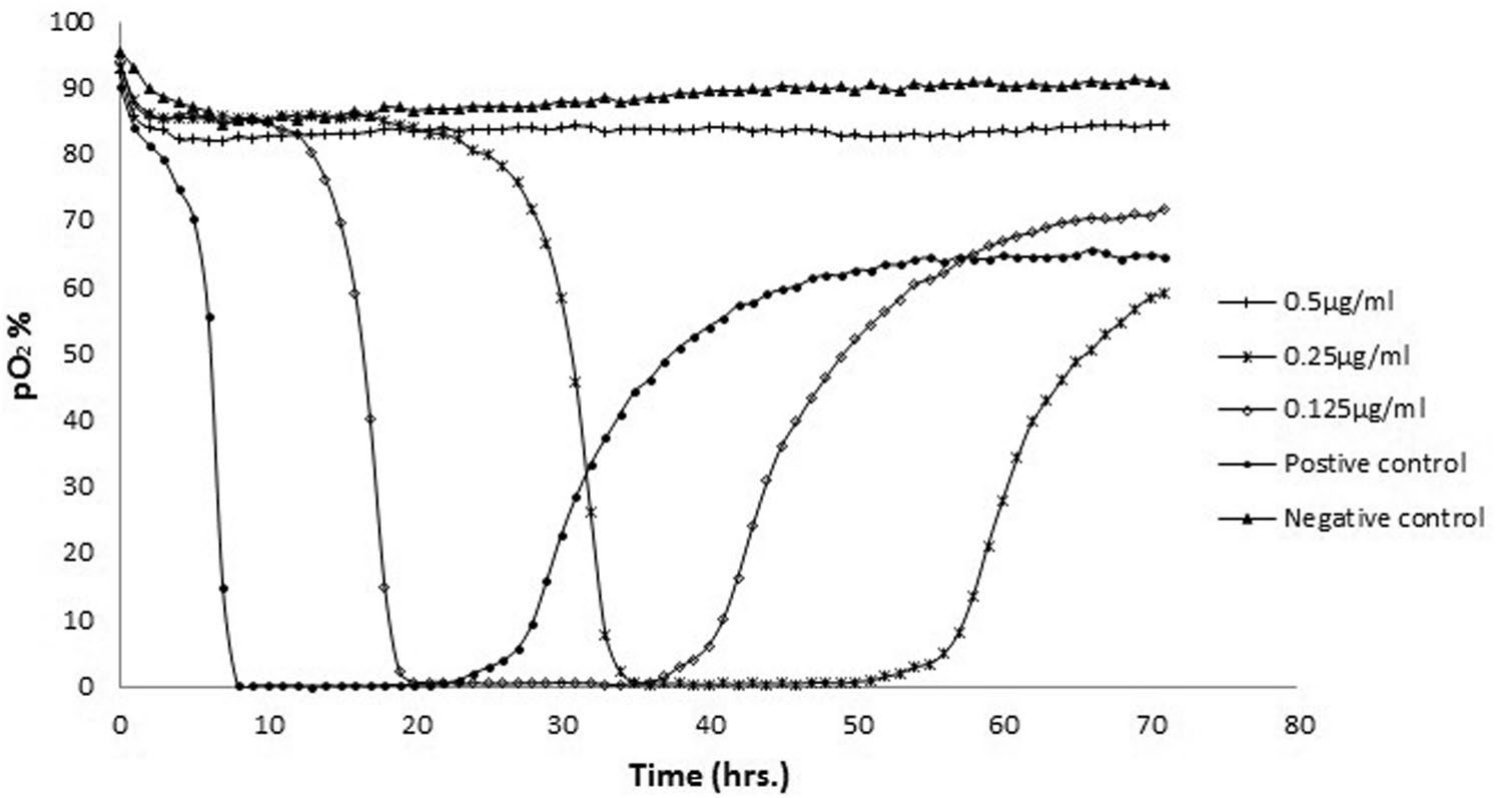
The MBC of tobramycin against PAO1*.* The oxygen consumption of an overnight culture of planktonic cells of PAO1 treated with tobramycin using a fluorescent plate reader incubated at 30 °C for 72 h. The MBC represents the time to detect the lowest concentration that induced an increase in the oxygen concentration as a result of oxygen diffusion back into the wells following cell death. In contrast to the untreated positive control sample, the negative control represents an overnight culture injected with solution of sodium hypochorite (10% v/v)

The MBC was determined using the OxoPlate reader on the basis of levels of oxygen depletion. The minimum antibiotic concentration reflecting the MBC was attained by plotting the oxygen concentration alongside time. The MBC was determined based on the re-introduction of atmospheric oxygen into the well once the cells had died, which caused the MBC correspondent to the minimum concentration to trigger a rise in the oxygen concentration. Every bacterial culture sample that antibiotics were applied to, was characterised by an oxygen depletion curve. Figures 1 and 2 indicate the MBCs of the strain that was tested. Amikacin had an MBC of 2.5 µg/ml, whereas tobramycin had an MBC of 0.5 µg/ml.

### Effect of divalent cation individually on the susceptibility of *P. aeruginosa* to amikacin and tobramycin

**Fig. 3:**
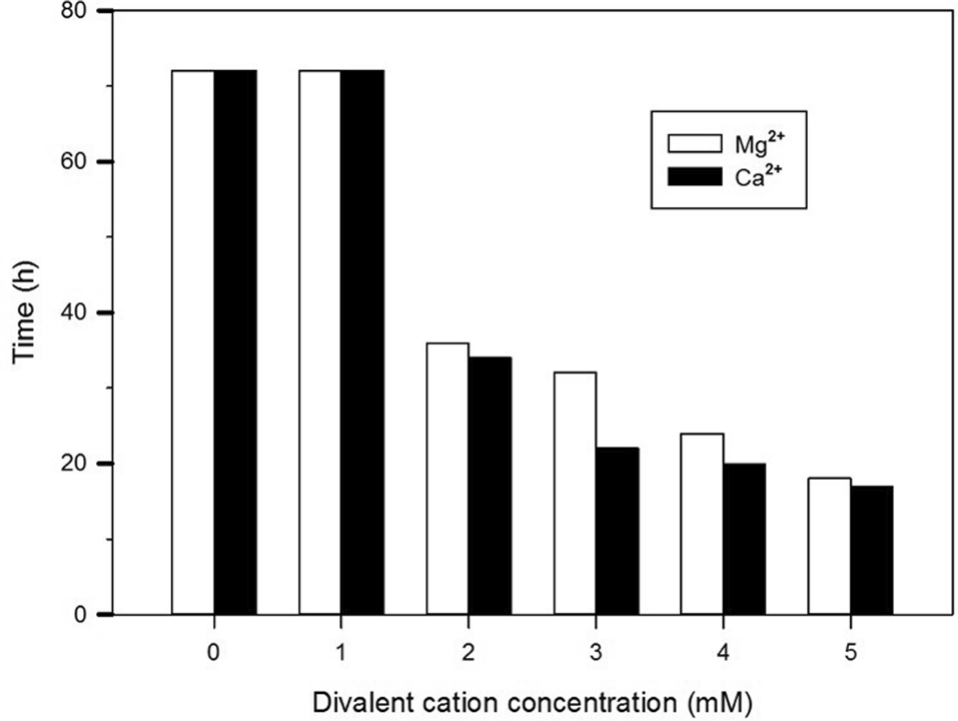
The effect of various concentrations of Mg^2+^ and Ca^2+^ on PA01 treated with amikacin at 2.5 µg ml^−1^. The amount of dissolved oxygen was followed over 72 h. The time represents the initial decrease of oxygen level “time to detect”. The values shown are the means of three replicates. Where “time to detect “values are shown as 72 h this indicates that no oxygen consumption was detected in the duration of the experiment

**Fig. 4:**
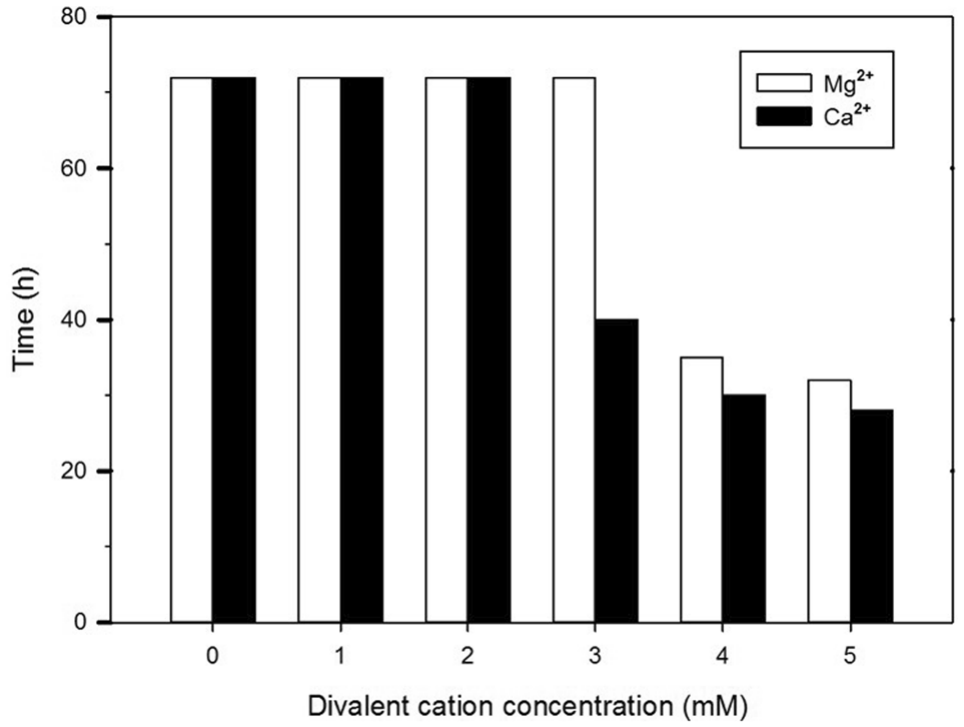
The effect of various concentrations of Mg^2+^ and Ca^2+^ on PA01 treated with tobramycin at 0.5 µg ml^−1^. The amount of dissolved oxygen was followed over 72 h. The time represents the initial decrease of oxygen level “time to detect”. The values shown are the means of three replicates. Where “time to detect” values are shown as 72 h this indicates that no oxygen consumption was detected in the duration of the experiment

The effect of the various concentrations of Mg^2+^or Ca^2+^ on the susceptibility of *P. aeruginosa* to lethal doses of the antibiotics is shown in Figs. 3 and 4. Reduced levels of pO_2_ indicate actively growing cells and that a decrease in pO2 show's active metabolism and an increase in pO_2_ indicate that cell metabolism is declining probably due to inhibition of growth by the higher concentration of ions. *P. aeruginosa* showed a change in the growth inhibition when the concentration of Ca^2+^or Mg^2+^ was increased. The consumption of oxygen was observed immediately at a high cation concentration whereas; oxygen consumption occurred at a slower rate at low cation concentrations.

The action of amikacin was gradually obliterated, shown by a decline in the duration of oxygen depletion as the concentration of Ca^2+^ and Mg^2+^ were increased from 2 to 5 mM (Fig. 3). Figure 4 illustrates that when the medium was treated with tobramycin in the presence of Mg^2+^ concentrations, ranging from 1 to 3 mM, no oxygen consumption occurred. This effect was seen in media where calcium was absent. The addition of ≥ 3 mM of Ca^2+^ in defined media resulted in microbial growth in the presence of tobramycin (Fig. 4), as seen as a gradual decrease in the oxygen. However, no oxygen consumption was observed to occur when a bactericidal dose of aminoglycoside was applied to *P. aeruginosa* in the absence of divalent cations. The study results corroborate that, in comparison to tobramycin, amikacin has a weaker effect on *P. aeruginosa* when divalent cations were individually added in defined media. Ca^2+^ was found to provide greater protection to bacterial cells over that provided by Mg^2+^.

### Effect of Mg^2+^ in combination with Ca^2+^ on the *in-vitro* susceptibility of *P*. *aeruginosa* to amikacin and tobramycin

The impact of combining Mg^2+^ and Ca^2+^ on the in vitro action of amikacin and tobramycin on *P. aeruginosa* was also investigated using the OxoPlate reader, which was based on the depletion of dissolved oxygen (Tables 1 and 2). It was observed that the combined concentrations of Mg^2+^ and Ca^2+^ had more of an impact on the effect of amikacin than that of tobramycin. The growth of *P. aeruginosa* was suppressed by tobramycin (0.5 µg/ml) for a long period time when 1 mM of Ca^2+^ was added in the defined medium in the presence of increasing concentrations of Mg^2+^ (2–5 mM) compared with amikacin. The duration of dissolved oxygen declined when the Mg^2+^ concentration was increased, from 72 h at 1 mM to 21 h at 5 mM (see Table 2). In contrast, the in vitro efficacy of amikacin against PA01 was reduced noticeably by the combined effects of Ca^2+^ and Mg^2+^supplementation. Table 1 shows that the period of growth inhibition declined when it was in the presence of a bactericidal concentration of amikacin at increasing concentrations of Mg^2+^ combined with a constant concentration of Ca^2+^ (1 mM), from 35 h at 1 mM to 13 h at 5 mM Mg^2+^.

**Table 1:** Effect of combinations of Mg^2+^ and Ca^2+^ on the rate of dissolved oxygen depletion in culture media treated with 2.5* µg* ml^−1^ amikacin

Time to detect in hrs. & divalent cations concentration mM shown in parenthesis					
	Mg^2+^(1)	Mg^2+^(2)	Mg^2+^(3)	Mg^2+^(4)	Mg^2+^(5)
Ca^2+^ (1)	35	28	17	15	13
Ca^2+^ (2)	18	16	14	13	12
Ca^2+^ (3)	13	13	11	11	11
Ca^2+^ (4)	12	12	11	11	10
Ca^2+^ (5)	12	11	10	10	10

The time represents the initial decrease of oxygen level “time to detect”. The values shown are the means of three replicates

**Table 2:** Effect of combinations of Mg^2+^ and Ca^2+^ on the rate of dissolved oxygen depletion in culture media treated with tobramycin at 0.5* µg* ml^−1^

Time to detect in hrs. & divalent cations concentration mM shown in parenthesis					
	Mg^2+^(1)	Mg^2+^(2)	Mg^2+^(3)	Mg^2+^(4)	Mg^2+^(5)
Ca^2+^ (1)	72*	51	32	25	21
Ca^2+^ (2)	42	31	27	19	18
Ca^2+^ (3)	26	19	16	15	14
Ca^2+^ (4)	17	16	15	15	14
Ca^2+^ (5)	14	14	14	14	14

The time represents the initial decrease of oxygen level “time to detect”. The values shown are the means of three replicates. *: means there was no oxygen depletion was detected over 72 h

The protection of bacteria cells against the action of antibiotics, provided by divalent cations, was visibly displayed. High concentrations of Ca^2+^ provided a greater level of protection when it was combined with Mg^2+^. The susceptibility of *P. aeruginosa* to the effects of antibiotics was low in media that contained high concentrations of Ca^2+^ combined with Mg^2+^. The bactericidal activity of amikacin was progressively reduced when concentrations of 4 or 5 mM of Ca^2+^ were added alongside increased concentrations of Mg^2+^ (see Table 1). The results show that the effects of Mg^2+^ were removed when combined with Ca^2+^ (5 mM). The depletion of dissolved oxygen was detected following a period of incubation of 14 h when *P. aeruginosa* treated with tobramycin in the present of Mg^2+^ concentrations, ranging from 1 to 5 mM combined with 5 mM of Ca^2+^ (Table 2).

## Discussion

This study has evaluated the use of a fluorescence-based assay to determine the oxygen concentration in the growth environment. The OxoPlate method has proven to be an accurate method to test the susceptibility of *P. aeruginosa* to antibiotics. To evaluate the correlation between the experimental results and studies published previously, the dissolved oxygen curves revealed that the extent to which *P. aeruginosa* was susceptible to antibiotics depends on the concentration of divalent cations in the test media. Similar results have been published previously based on the susceptibility of *P. aeruginosa* to both aminoglycosides and colistin in present of the cations composition of broth as well as agar (Jeffrey et al. 1982). Posing a major issue in aminoglycoside therapy, aminoglycoside antagonism by divalent cations has been comprehensively explored in the case of *P. aeruginosa* (Medeiros et al. 1971; Zimelis and Jackson 1973; D’Amato et al. 1975). As confirmed by many studies, this antagonism is caused by the fact that aminoglycoside assimilation at sites on the external and internal membrane is disrupted by cations (Bryan and Van De Elzen 1977; Campbell and Kadner 1980; Zimelis and Jackson 1973). Additional evidence in support of the fact that the internal membrane is a site for cation antagonism was provided by Mao et al. (2001), which demonstrated that aminoglycoside assimilation in *Staphylococcus aureus* and in spheroplasts of *Escherichia coli* seemed to be suppressed by cations. Furthermore, according to the findings of Mao et al. (2001), a direct correlation exists between the functionality of the MexXY-OprM efflux pump and antagonism of aminoglycoside by divalent cations.

Based on the oxygen depletion curves, Mg^2+^ was not as effective as Ca^2+^ in suppressing the effect of aminoglycoside agents against *P. aeruginosa* (Figs. 3–4). Similar results were found in a recent study involving Gram negative bacteria (*E. coli*) (Sahalan et al. 2013), where the activity of Polymyxin B was significantly inhibited in the present of Ca^2+^ in defined media, which lead to survival bacteria. The experimental findings suggest that the greater efficiency of Ca^2+^ can be explained in terms of specific cations adapted to particular functions. In fact, Ca^2+^ is rather an element of the cell structural components, and less frequently a co-factor of active proteins (bacterial Complex I) as Mg^2+^in *E. coli* (Verkhovskaya, Knuuti, and Wikström 2011). The reliability of the observation of growth inhibitors is enhanced by the oxygen consumption measurements using Oxoplate. At present, the sensitivity of viable cells of bacteria and mammalian cells can be measured through several assays such as formazan dye reduction (resazurin). Resazurin is commonly used as an indicator for screening drug interactions (Kuete et al. 2017). Compared with tetrazolium assays, the resazurin reduction method is much more sensitive and economical (Riss et al. 2013). However, fluorescence-based methods are more sensitive than absorbance-based methods, such as the resazurin assays, where the blue dye is oxidised to pink by living cells.

## Conclusion

It can be concluded that the kinetics of growth suppression of any aerobic microorganism can be evaluated with the microplate-based system containing flurophores sensitive to oxygen. Compared to standard methods, the OxoPlate represents a viable, flexible and straightforward option for undertaking bacterial susceptibility assessments in broth as it has been demonstrated to be a fast, automated technique (although its cost is not trivial). In addition, this assay permits a high sample throughout application and displays sensitivity, reproducibility and robustness. These advantages make the system appropriate for high-content assays in most fields of microbiological research. Moreover, the OxoPlate also facilitates the preliminary characterisation of antibiotics susceptibility. This study has explored the extent to which the susceptibility of *P. aeruginosa* to aminoglycosides is affected by different concentrations of Ca^2+^ and Mg^2+^. Based on the experiment conducted, it has been possible to derive certain observations regarding the competition between the two divalent cations. The experimental results revealed that the susceptibility of *P. aeruginosa* to antibiotics is influenced by both divalent cations. Furthermore, since Ca^2+^ is a more frequent element of bacterial structural components, its efficiency in providing protection to bacterial cells is greater than that of Mg^2+^. Therefore, when conducting susceptibility testing on any species of *Pseudomonas*, the influence of these cations should be taken into account.
